# Structural data of thermostable 3D Ln-MOFs that based on flexible ligand of 1,3-adamantanediacetic acid

**DOI:** 10.1016/j.dib.2018.01.094

**Published:** 2018-02-03

**Authors:** Cheng-Hui Zeng, Hao-Ran Li, Zi-Qi Liu, Fei Chen, Shengliang Zhong

**Affiliations:** College of Chemistry and Chemical Engineering, Jiangxi Normal University, Nanchang 330022, PR China

## Abstract

In this data article, we present the structural and PARD data of the Ln-MOFs. Detailed structure, luminescence and sensing properties were discussed in our previous study (Zeng et al., in press) [1] The data includes the SBU structure patterns of these Ln-MOFs, thermostability of Ln-MOFs in water and also detailed structure information listed in Table 1, Table 2, Table 3, Table 4, Table 5, Table 6, Table 7, Table 8.

**Specifications Table** [Please fill in right-hand column of the table below.]

**Table:** 

Subject area	*Chemistry*
More specific subject area	*Single crystal data of lanthanide complexes*
Type of data	*Table, figure*
How data was acquired	*Crystallography open data base and crystallographic tool – Diamond: Crystallographic Information File Code: 1562078–1562085.cif*
Data format	*Analyzed*
Experimental factors	*Single crystal X-ray diffraction data was collected on a Bruker SMART 1000 CCD at 293(2) K, with Mo-Ka radiation (0.71073Å) at room temperature. The structure was refined by full-matrix least-squares methods with SHELXL-97 module. The eight structures are isostructural, they crystallize in monoclinic space group C2/c (no. 15).*
Experimental features	*Needle like colorless single crystal.*
Data source location	*Jiangxi Normal University, Nanchang, China.*
Data accessibility	*The data are with this article.*
Related research article	*K. Zheng, Z.-Q. Liu, Y. Huang, F. Chen, C.-H. Zeng, S. Zhong, et al., Highly Luminescent Ln-MOFs Based on 1,3-Adamantanediacetic Acid as Bifunctional Sensor, Sensors and Actuators B: Chemical, in press.*

**Value of the data**

• This data would be valuable for further studies of lanthanide complexes that based on flexible ligand.
• This data would be valuable for the further studies of lanthanide complexes that coordinated by phen.
• This data provide a new way to synthesize thermostable lanthanide complexes.

## 1 Data

**Fig. 1:**
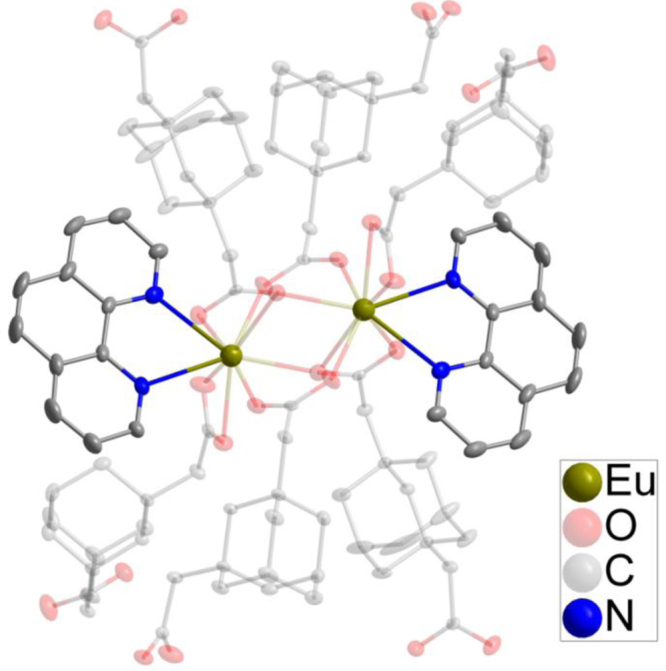
The SBU structure shows the coordination environment of the metal ions, two chelated phen arranged at two ends of the dinuclear cluster.

**Fig. 2:**
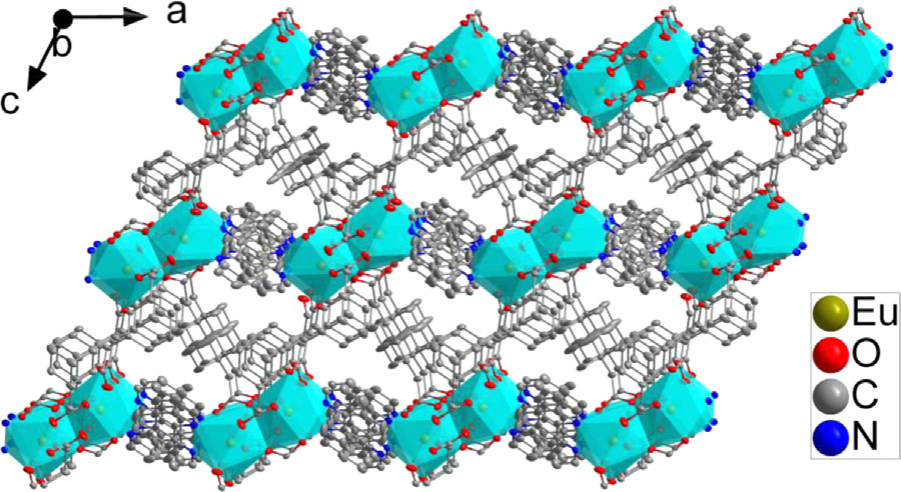
The 3D Ln-MOFs structure of **1a** view from the ob direction.

**Fig. 3:**
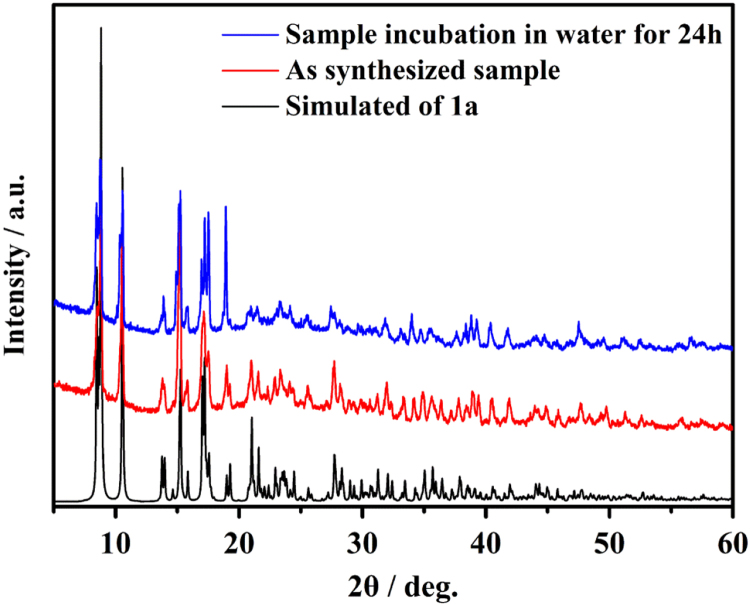
PXRD patterns comparison of single crystal data **1a**, as synthesized **1a** and bulk sample **1a** immersed in water for 24 h, they compete with each other very well, confirming **1a** is highly stable that incubated in aqueous solution for 24 h.

The crystal structures of isostructural **1a**-**1h** have the same chemical formula of [Ln(ADA)_1.5_(phen)]_n_ (Ln^3+^ = Eu^3+^ **1a**, Gd^3+^ **1b**, Tb^3+^ **1c**, La^3+^ **1d**, Ce^3+^ **1e**, Pr^3+^ **1f**, Nd^3+^ **1g**, Y^3+^ **1h**, 1,10-phenanthroline = phen) [1]*. Since they are isostructural data, as an example, the crystal structure of **1a** is described in somewhat greater detail.* As shown in Fig. 1, two crystallographically independent Eu^3+^ are bridged by two carboxyl and two O, each dinuclear second building unit (SBU) contains two Eu^3+^, two phen and three fully deprotonated ADA, forming a electroneutral unit. Two phen arranged at two ends of the dinuclear cluster (Fig. 2). The coordination environment of the nine-coordinated Eu^3+^ center consists seven O and two N. Detailed information about selected bond lengths and angles for **1a**–**1h** are listed in Table 1, Table 2, Table 3, Table 4, Table 5, Table 6, Table 7, Table 8, they show that the bond lengths and angles are in the normal value as our previous reports [2], [3], [4], [5], [6], [7], [8], [9]. PXRD patterns of **1a** that incubation in H_2_O for 24 h competes well with the simulated single crystal data and the as synthesized sample, confirming **1a** has high stability in water and also has high stability while sensing (Fig. 3).

**Table 1:** Selected bond lengths and bond angles of **1a**.

Eu(1)-O(1)	2.3751(14)	Eu(1)-N(2)	2.5961(16)
Eu(1)-O(2)	2.3779(13)	Eu(1)-N(1)	2.6563(16)
Eu(1)-O(4)	2.3802(14)	Eu(1)-O(4)#1	2.7171(15)
Eu(1)-O(8)	2.4397(14)	Eu(1)-C(14)	2.8253(19)
Eu(1)-O(7)	2.4749(14)	Eu(1)-C(15)	2.9887(19)
Eu(1)-O(3)	2.4859(14)	Eu(1)-Eu(1)#1	3.9681(2)
O(1)-Eu(1)-O(2)	136.90(5)	O(1)-Eu(1)-O(4)#1	76.74(5)
O(1)-Eu(1)-O(4)	73.03(5)	O(2)-Eu(1)-O(4)#1	97.21(5)
O(2)-Eu(1)-O(4)	75.65(5)	O(4)-Eu(1)-O(4)#1	165.75(5)
O(1)-Eu(1)-O(8)	127.43(5)	O(8)-Eu(1)-O(4)#1	87.00(5)
O(2)-Eu(1)-O(8)	75.10(5)	O(7)-Eu(1)-O(4)#1	71.15(5)
O(4)-Eu(1)-O(8)	81.74(5)	O(3)-Eu(1)-O(4)#1	101.67(5)
O(1)-Eu(1)-O(7)	83.91(5)	N(2)-Eu(1)-O(4)#1	143.89(5)
O(2)-Eu(1)-O(7)	128.06(5)	N(1)-Eu(1)-O(4)#1	157.58(5)
O(4)-Eu(1)-O(7)	95.22(5)	O(1)-Eu(1)-C(14)	24.14(5)
O(8)-Eu(1)-O(7)	52.96(5)	O(2)-Eu(1)-C(14)	89.84(5)
O(1)-Eu(1)-O(3)	102.46(5)	O(4)-Eu(1)-C(14)	80.90(5)
O(2)-Eu(1)-O(3)	71.88(5)	O(8)-Eu(1)-C(14)	25.13(5)
O(4)-Eu(1)-O(3)	123.75(5)	O(7)-Eu(1)-C(14)	165.57(5)
O(8)-Eu(1)-O(3)	129.69(5)	O(3)-Eu(1)-C(14)	72.15(5)
O(7)-Eu(1)-O(3)	140.83(5)	N(2)-Eu(1)-C(14)	72.95(4)
O(1)-Eu(1)-N(2)	135.35(5)	N(1)-Eu(1)-C(14)	77.96(5)
O(2)-Eu(1)-N(2)	82.69(5)	O(4)#1-Eu(1)-C(14)	145.45(5)
O(4)-Eu(1)-N(2)	150.43(5)	O(1)-Eu(1)-C(15)	156.06(5)
O(8)-Eu(1)-N(2)	73.32(5)	O(2)-Eu(1)-C(15)	49.26(4)
O(7)-Eu(1)-N(2)	82.42(5)	O(4)-Eu(1)-C(15)	114.73(5)
O(3)-Eu(1)-N(2)	65.81(5)	O(8)-Eu(1)-C(15)	95.79(5)
O(1)-Eu(1)-N(1)	73.00(5)	O(7)-Eu(1)-C(15)	106.22(6)
O(2)-Eu(1)-N(1)	135.22(5)	O(3)-Eu(1)-C(15)	101.45(5)
O(4)-Eu(1)-N(1)	145.73(5)	N(2)-Eu(1)-C(15)	87.99(5)
O(8)-Eu(1)-N(1)	116.30(5)	N(1)-Eu(1)-C(15)	26.35(5)
O(7)-Eu(1)-N(1)	76.85(5)	O(4)#1-Eu(1)-C(15)	26.61(5)
O(3)-Eu(1)-N(1)	68.60(5)	C(14)-Eu(1)-C(15)	142.43(5)
N(2)-Eu(1)-N(1)	62.54(5)		

Symmetry transformations used to generate equivalent atoms: #1 -x+1/2, -y+3/2, -z; #2 -x, y, -z-1/2; #3 -x+1/2, y+1/2, -z+1/2; #4 -x+1/2, y-1/2, -z+1/2.

**Table 2:** Selected bond lengths and bond angles of **1b**.

Gd(1)-O(2)#1	2.451(3)	Gd(1)-N(1)	2.675(3)
Gd(1)-O(1)	2.455(3)	Gd(1)-N(2)	2.732(3)
Gd(1)-O(6)#1	2.463(3)	Gd(1)-O(6)	2.751(3)
Gd(1)-O(4)#2	2.506(3)	Gd(1)-C(14)#2	2.900(3)
Gd(1)-O(3)#2	2.554(3)	Gd(1)-C(15)	3.041(3)
Gd(1)-O(5)	2.567(3)	Gd(1)-Gd(1)#1	4.0419(4)
O(2)#1-Gd(1)-O(1)	136.41(9)	N(1)-Gd(1)-C(14)#2	76.19(9)
O(2)#1-Gd(1)-O(6)#1	74.81(9)	N(2)-Gd(1)-C(14)#2	97.41(10)
O(1)-Gd(1)-O(6)#1	72.69(9)	O(6)-Gd(1)-C(14)#2	167.91(9)
O(2)#1-Gd(1)-O(4)#2	75.64(9)	O(2)#1-Gd(1)-C(15)	70.67(9)
O(1)-Gd(1)-O(4)#2	127.59(10)	O(1)-Gd(1)-C(15)	88.25(10)
O(6)#1-Gd(1)-O(4)#2	83.65(9)	O(6)#1-Gd(1)-C(15)	101.55(9)
O(2)#1-Gd(1)-O(3)#2	127.00(9)	O(4)#2-Gd(1)-C(15)	142.99(9)
O(1)-Gd(1)-O(3)#2	85.20(9)	O(3)#2-Gd(1)-C(15)	157.89(9)
O(6)#1-Gd(1)-O(3)#2	96.61(10)	O(5)-Gd(1)-C(15)	23.77(9)
O(4)#2-Gd(1)-O(3)#2	51.36(9)	O(2)#1-Gd(1)-O(6)	72.66(8)
O(2)#1-Gd(1)-O(5)	71.06(9)	O(1)-Gd(1)-O(6)	73.03(9)
O(1)-Gd(1)-O(5)	104.02(10)	O(6)#1-Gd(1)-O(6)	78.49(9)
O(6)#1-Gd(1)-O(5)	122.94(9)	O(4)#2-Gd(1)-O(6)	146.69(9)
O(4)#2-Gd(1)-O(5)	127.86(9)	O(3)#2-Gd(1)-O(6)	158.18(9)
O(3)#2-Gd(1)-O(5)	140.43(9)	O(5)-Gd(1)-O(6)	48.43(8)
O(2)#1-Gd(1)-N(1)	84.53(10)	N(1)-Gd(1)-O(6)	112.97(8)
O(1)-Gd(1)-N(1)	133.98(10)	N(2)-Gd(1)-O(6)	94.09(8)
O(6)#1-Gd(1)-N(1)	152.34(9)	O(2)#1-Gd(1)-C(14)#2	101.34(10)
O(4)#2-Gd(1)-N(1)	73.40(9)	O(1)-Gd(1)-C(14)#2	106.80(10)
O(3)#2-Gd(1)-N(1)	81.15(10)	O(6)#1-Gd(1)-C(14)#2	89.86(9)
O(5)-Gd(1)-N(1)	64.63(9)	O(4)#2-Gd(1)-C(14)#2	25.70(10)
O(2)#1-Gd(1)-N(2)	135.25(10)	O(3)#2-Gd(1)-C(14)#2	25.66(10)
O(1)-Gd(1)-N(2)	73.11(10)	O(5)-Gd(1)-C(14)#2	140.48(9)
O(6)#1-Gd(1)-N(2)	145.69(10)	N(1)-Gd(1)-C(15)	88.38(9)
O(4)#2-Gd(1)-N(2)	115.87(9)	N(2)-Gd(1)-C(15)	79.98(9)
O(3)#2-Gd(1)-N(2)	77.91(10)	O(6)-Gd(1)-C(15	24.68(9)
O(5)-Gd(1)-N(2)	68.67(9)	C(14)#2-Gd(1)-C(15)	163.41(10)
N(1)-Gd(1)-N(2)	61.12(10)		

Symmetry transformations used to generate equivalent atoms: #1 -x+1, -y, -z+1; #2 ×+1/2, -y+1/2, z+1/2; #3 -x+1, y, -z+1/2; #4 ×-1/2, -y+1/2, z-1/2.

**Table 3:** Selected bond lengths and bond angles of **1c**.

Tb(1)-O(6)	2.3454(17)	Tb(1)-N(1)	2.5730(18)
Tb(1)-O(2)	2.3457(16)	Tb(1)-N(2)	2.6375(18)
Tb(1)-O(1)	2.3561(15)	Tb(1)-O(6)#2	2.775(2)
Tb(1)-O(3)#1	2.4232(15)	Tb(1)-C(14)#1	2.803(2)
Tb(1)-O(4)#1	2.4499(16)	Tb(1)-C(15)	3.004(2)
Tb(1)-O(5)	2.4514(16)	Tb(1)-Tb(1)#2	3.9815(2)
O(6)-Tb(1)-O(2)	73.26(6)	O(5)-Tb(1)-N(2)	68.73(6)
O(6)-Tb(1)-O(1)	76.26(6)	N(1)-Tb(1)-N(2)	63.11(6)
O(2)-Tb(1)-O(1)	136.15(5)	N(1)-Tb(1)-C(14)#1	77.04(6)
O(6)-Tb(1)-O(3)#1	81.08(6)	N(2)-Tb(1)-C(14)#1	97.21(6)
O(2)-Tb(1)-O(3)#1	128.82(6)	O(6)#2-Tb(1)-C(14)#1	164.94(6)
O(1)-Tb(1)-O(3)#1	75.06(5)	O(6)-Tb(1)-C(15)	101.89(6)
O(6)-Tb(1)-O(4)#1	94.30(7)	O(2)-Tb(1)-C(15)	85.32(6)
O(2)-Tb(1)-O(4)#1	84.85(6)	O(1)-Tb(1)-C(15)	70.95(5)
O(1)-Tb(1)-O(4)#1	128.48(5)	O(3)#1-Tb(1)-C(15)	143.93(6)
O(3)#1-Tb(1)-O(4)#1	53.41(5)	O(4)#1-Tb(1)-C(15)	157.75(6)
O(6)-Tb(1)-O(5)	124.00(6)	O(5)-Tb(1)-C(15)	23.83(5)
O(2)-Tb(1)-O(5)	100.16(6)	O(6)-Tb(1)-O(6)#2	78.25(6)
O(1)-Tb(1)-O(5)	72.18(6)	O(2)-Tb(1)-O(6)#2	71.34(6)
O(3)#1-Tb(1)-O(5)	130.71(6)	O(1)-Tb(1)-O(6)#2	72.17(5)
O(4)#1-Tb(1)-O(5)	141.35(6)	O(3)#1-Tb(1)-O(6)#2	144.49(5)
O(6)-Tb(1)-N(1)	149.90(6)	O(4)#1-Tb(1)-O(6)#2	156.17(5)
O(2)-Tb(1)-N(1)	135.76(6)	O(5)-Tb(1)-O(6)#2	48.61(5)
O(1)-Tb(1)-N(1)	82.12(6)	N(1)-Tb(1)-O(6)#2	114.75(5)
O(3)#1-Tb(1)-N(1)	73.21(6)	N(2)-Tb(1)-O(6)#2	96.65(5)
O(4)#1-Tb(1)-N(1)	82.92(6)	O(6)-Tb(1)-C(14)#1	86.99(6)
O(5)-Tb(1)-N(1)	66.72(5)	O(2)-Tb(1)-C(14)#1	107.40(6)
O(6)-Tb(1)-N(2)	145.39(6)	O(1)-Tb(1)-C(14)#1	101.62(6)
O(2)-Tb(1)-N(2)	72.71(6)	O(3)#1-Tb(1)-C(14)#1	26.56(6)
O(1)-Tb(1)-N(2)	135.27(6)	O(4)#1-Tb(1)-C(14)#1	26.86(6)
O(3)#1-Tb(1)-N(2)	116.39(6)	O(5)-Tb(1)-C(14)#1	143.70(6)
O(4)#1-Tb(1)-N(2)	76.57(6)		

Symmetry transformations used to generate equivalent atoms: #1 -x+1/2, y+1/2, -z+1/2; #2 -x+1/2, -y+1/2, -z; #3 -x, y, -z-1/2; #4 -x+1/2, y-1/2, -z+1/2.

**Table 4:** Selected bond lengths and bond angles of **1d**.

La(1)-O(3)	2.4706(13)	La(1)-N(1)	2.6995(16)
La(1)-O(4)#1	2.4786(14)	La(1)-N(2)	2.7534(16)
La(1)-O(5)	2.4866(13)	La(1)-O(5)#1	2.7712(13)
La(1)-O(2)	2.5295(13)	La(1)-C(1)	2.9250(18)
La(1)-O(1)	2.5790(14)	La(1)-C(15)#1	3.0650(18)
La(1)-O(6)#1	2.5869(13)	La(1)-La(1)#1	4.0771(2)
O(3)-La(1)-O(4)#1	135.96(4)	N(1)-La(1)-C(1)	76.01(5)
O(3)-La(1)-O(5)	74.67(5)	N(2)-La(1)-C(1)	97.56(5)
O(4)#1-La(1)-O(5)	72.44(5)	O(5)#1-La(1)-C(1)	168.33(5)
O(3)-La(1)-O(2)	75.84(5)	O(3)-La(1)-C(15)#1	70.48(5)
O(4)#1-La(1)-O(2)	128.06(5)	O(4)#1-La(1)-C(15)#1	88.19(5)
O(5)-La(1)-O(2)	84.42(4)	O(5)-La(1)-C(15)#1	101.37(5)
O(3)-La(1)-O(1)	126.80(4)	O(2)-La(1)-C(15)#1	142.69(5)
O(4)#1-La(1)-O(1)	85.97(5)	O(1)-La(1)-C(15)#1	157.86(5)
O(5)-La(1)-O(1)	97.18(5)	O(6)#1-La(1)-C(15)#1	23.55(4)
O(2)-La(1)-O(1)	50.96(5)	O(3)-La(1)-O(5)#1	72.35(4)
O(3)-La(1)-O(6)#1	70.99(5)	O(4)#1-La(1)-O(5)#1	73.02(5)
O(4)#1-La(1)-O(6)#1	103.85(5)	O(5)-La(1)-O(5)#1	78.45(4)
O(5)-La(1)-O(6)#1	122.60(4)	O(2)-La(1)-O(5)#1	146.84(4)
O(2)-La(1)-O(6)#1	127.53(5)	O(1)-La(1)-O(5)#1	158.92(4)
O(1)-La(1)-O(6)#1	140.21(5)	O(6)#1-La(1)-O(5)#1	48.05(4)
O(3)-La(1)-N(1)	85.16(5)	N(1)-La(1)-O(5)#1	112.44(4)
O(4)#1-La(1)-N(1)	133.54(5)	N(2)-La(1)-O(5)#1	93.75(4)
O(5)-La(1)-N(1)	153.15(5)	O(3)-La(1)-C(1)	101.23(5)
O(2)-La(1)-N(1)	73.42(5)	O(4)#1-La(1)-C(1)	107.49(5)
O(1)-La(1)-N(1)	80.71(5)	O(5)-La(1)-C(1)	90.52(5)
O(6)#1-La(1)-N(1)	64.45(4)	O(2)-La(1)-C(1)	25.40(5)
O(3)-La(1)-N(2)	135.03(5)	O(1)-La(1)-C(1)	25.56(5)
O(4)#1-La(1)-N(2)	73.37(5)	O(6)#1-La(1)-C(1)	140.04(5)
O(5)-La(1)-N(2)	145.73(5)	N(1)-La(1)-C(15)#1	87.99(5)
O(2)-La(1)-N(2)	115.65(5)	N(2)-La(1)-C(15)#1	79.68(5)
O(1)-La(1)-N(2)	78.19(5)		

Symmetry transformations used to generate equivalent atoms: #1 -x+3/2, -y+3/2, -z+1; #2 -x+3/2, y+1/2, -z+3/2; #3 -x+2, y, -z+3/2; #4 -x+3/2, y-1/2, -z+3/2.

**Table 5:** Selected bond lengths and bond angles of **1e**.

Ce(1)-O(1)	2.4498(14)	Ce(1)-N(1)	2.6756(17)
Ce(1)-O(2)#1	2.4536(15)	Ce(1)-N(2)	2.7296(17)
Ce(1)-O(6)#1	2.4653(14)	Ce(1)-O(6)	2.7514(14)
Ce(1)-O(3)#2	2.5054(14)	Ce(1)-C(14)#2	2.9004(19)
Ce(1)-O(4)#2	2.5545(15)2.	Ce(1)-C(15)	3.0425(19)
Ce(1)-O(5)	5674(14)	Ce(1)-Ce(1)#1	4.0437(2)
O(1)-Ce(1)-O(2)#1	136.41(5)	O(1)-Ce(1)-O(6)	76.15(5)
O(1)-Ce(1)-O(6)#1	74.85(5)	O(2)#1-Ce(1)-O(6)	97.46(6)
O(2)#1-Ce(1)-O(6)#1	72.59(5)	O(6)#1-Ce(1)-O(6)	167.96(5)
O(1)-Ce(1)-O(3)#2	75.48(5)	O(3)#2-Ce(1)-O(6)	70.78(5)
O(2)#1-Ce(1)-O(3)#2	127.72(5)	O(4)#2-Ce(1)-O(6)	88.20(5)
O(6)#1-Ce(1)-O(3)#2	83.75(5)	O(5)-Ce(1)-O(6)	101.56(5)
O(1)-Ce(1)-O(4)#2	126.91(5)	N(1)-Ce(1)-O(6)	142.91(5)
O(2)#1-Ce(1)-O(4)#2	85.32(5)	N(2)-Ce(1)-O(6)	157.77(5)
O(6)#1-Ce(1)-O(4)#2	96.74(5)	O(1)-Ce(1)-C(14)#2	72.76(4)
O(3)#2-Ce(1)-O(4)#2	51.43(5)	O(2)#1-Ce(1)-C(14)#2	72.98(5)
O(1)-Ce(1)-O(5)	71.15(5)	O(6)#1-Ce(1)-C(14)#2	78.51(5)
O(2)#1-Ce(1)-O(5)	103.97(5)	O(3)#2-Ce(1)-C(14)#2	146.67(5)
O(6)#1-Ce(1)-O(5)	122.94(5)	O(4)#2-Ce(1)-C(14)#2	158.25(5)
O(3)#2-Ce(1)-O(5)	127.78(5)	O(5)-Ce(1)-C(14)#2	48.39(4)
O(4)#2-Ce(1)-O(5)	140.29(5)	N(1)-Ce(1)-C(14)#2	112.91(5)
O(1)-Ce(1)-N(1)	84.57(5)	N(2)-Ce(1)-C(14)#2	94.04(5)
O(2)#1-Ce(1)-N(1)	133.93(5)	O(6)-Ce(1)-C(14)#2	101.17(6)
O(6)#1-Ce(1)-N(1)	152.49(5)	O(1)-Ce(1)-C(15)	106.94(6)
O(3)#2-Ce(1)-N(1)	73.40(5)	O(2)#1-Ce(1)-C(15)	89.93(5)
O(4)#2-Ce(1)-N(1)	81.03(5)	O(6)#1-Ce(1)-C(15)	25.70(6)
O(5)-Ce(1)-N(1)	64.60(5)	O(3)#2-Ce(1)-C(15)	25.73(6)
O(1)-Ce(1)-N(2)	135.09(5)	O(4)#2-Ce(1)-C(15)	140.41(5)
O(2)#1-Ce(1)-N(2)	73.33(5)	O(5)-Ce(1)-C(15)	23.74(5)
O(6)#1-Ce(1)-N(2)	145.83(5)		

Symmetry transformations used to generate equivalent atoms: #1 -x+3/2, -y+3/2, -z+1; #2 -x+3/2, y+1/2, -z+3/2; #3 -x+2, y, -z+3/2; #4 -x+3/2, y-1/2, -z+3/2.

**Table 6:** Selected bond lengths and bond angles of **1f**.

Pr(1)-O(4)#1	2.4320(14)	Pr(1)-N(2)	2.6541(17)
Pr(1)-O(3)#2	2.4372(15)	Pr(1)-N(1)	2.7111(18)
Pr(1)-O(5)	2.4470(15)	Pr(1)-O(5)#3	2.7383(15)
Pr(1)-O(1)	2.4905(15)	Pr(1)-C(1)	2.883(2)
Pr(1)-O(2)	2.5366(15)	Pr(1)-C(15)#3	3.029(2)
Pr(1)-O(6)#3	2.5482(15)	Pr(1)-Pr(1)#3	4.0220(2)
O(4)#1-Pr(1)-O(3)#2	136.70(5)	O(4)#1-Pr(1)-O(5)#3	72.92(5)
O(4)#1-Pr(1)-O(5)	75.03(5)	O(3)#2-Pr(1)-O(5)#3	72.90(5)
O(3)#2-Pr(1)-O(5)	72.75(5)	O(5)-Pr(1)-O(5)#3	78.42(5)
O(4)#1-Pr(1)-O(1)	75.39(5)	O(1)-Pr(1)-O(5)#3	146.48(5)
O(3)#2-Pr(1)-O(1)	127.39(6)	O(2)-Pr(1)-O(5)#3	157.62(5)
O(5)-Pr(1)-O(1)	83.16(5)	O(6)#3-Pr(1)-O(5)#3	48.71(4)
O(4)#1-Pr(1)-O(2)	127.15(5)	N(2)-Pr(1)-O(5)#3	113.45(5)
O(3)#2-Pr(1)-O(2)	84.74(5)	N(1)-Pr(1)-O(5)#3	94.40(5)
O(5)-Pr(1)-O(2)	96.29(6)	O(4)#1-Pr(1)-C(1)	101.18(6)
O(1)-Pr(1)-O(2)	51.76(5)	O(3)#2-Pr(1)-C(1)	106.59(6)
O(4)#1-Pr(1)-O(6)#3	71.35(5)	O(5)-Pr(1)-C(1)	89.43(5)
O(3)#2-Pr(1)-O(6)#3	103.83(5)	O(1)-Pr(1)-C(1)	25.79(6)
O(5)-Pr(1)-O(6)#3	123.24(5)	O(2)-Pr(1)-C(1)	25.97(6)
O(1)-Pr(1)-O(6)#3	128.26(5)	O(6)#3-Pr(1)-C(1)	140.79(5)
O(2)-Pr(1)-O(6)#3	140.42(5)	N(2)-Pr(1)-C(1)	76.22(5)
O(4)#1-Pr(1)-N(2)	84.20(5)	N(1)-Pr(1)-C(1)	97.41(6)
O(3)#2-Pr(1)-N(2)	134.16(6)	O(5)#3-Pr(1)-C(1)	167.47(5)
O(5)-Pr(1)-N(2)	152.00(5)	O(4)#1-Pr(1)-C(15)#3	70.91(5)
O(1)-Pr(1)-N(2)	73.44(5)	O(3)#2-Pr(1)-C(15)#3	88.11(5)
O(2)-Pr(1)-N(2)	81.33(6)	O(5)-Pr(1)-C(15)#3	101.63(5)
O(6)#3-Pr(1)-N(2)	64.86(5)	O(1)-Pr(1)-C(15)#3	143.22(5)
O(4)#1-Pr(1)-N(1)	135.14(6)	O(2)-Pr(1)-C(15)#3	157.77(5)
O(3)#2-Pr(1)-N(1)	73.21(6)	O(6)#3-Pr(1)-C(15)#3	23.91(5)
O(5)-Pr(1)-N(1)	145.82(5)	N(2)-Pr(1)-C(15)#3	88.75(5)
O(1)-Pr(1)-N(1)	115.98(5)	N(1)-Pr(1)-C(15)#3	80.10(5)
O(2)-Pr(1)-N(1)	77.69(5)	O(5)#3-Pr(1)-C(15)#3	24.82(5)
O(6)#3-Pr(1)-N(1)	68.50(5)	C(1)-Pr(1)-C(15)#3	163.82(6)
N(2)-Pr(1)-N(1)	61.21(6)		

Symmetry transformations used to generate equivalent atoms: #1 -x+3/2, y-1/2, -z+3/2; #2 ×, -y+1, z-1/2; #3 -x+3/2, -y+1/2, -z+1; #4 -x+2, y, -z+3/2; #5 -x+3/2, y+1/2, -z+3/2; #6 ×, -y+1, z+1/2.

**Table 7:** Selected bond lengths and bond angles of **1 g**.

Nd(1)-O(1)	2.4192(14)	Nd(1)-N(2)	2.6360(18)
Nd(1)-O(2)#1	2.4211(15)	Nd(1)-N(1)	2.6923(18)
Nd(1)-O(5)#1	2.4298(15)	Nd(1)-O(5)	2.7278(16)
Nd(1)-O(4)#2	2.4757(15)	Nd(1)-C(14)#2	2.871(2)
Nd(1)-O(3)#2	2.5194(15)	Nd(1)-C(15)	3.015(2)
Nd(1)-O(6)	2.5307(15)	Nd(1)-Nd(1)#1	4.0061(2)
O(1)-Nd(1)-O(2)#1	136.86(5)	O(1)-Nd(1)-O(5)	73.02(5)
O(1)-Nd(1)-O(5)#1	75.10(5)	O(2)#1-Nd(1)-O(5)	72.83(5)
O(2)#1-Nd(1)-O(5)#1	72.81(5)	O(5)#1-Nd(1)-O(5)	78.23(5)
O(1)-Nd(1)-O(4)#2	75.31(5)	O(4)#2-Nd(1)-O(5)	146.28(5)
O(2)#1-Nd(1)-O(4)#2	127.15(6)	O(3)#2-Nd(1)-O(5)	157.21(5)
O(5)#1-Nd(1)-O(4)#2	82.78(5)	O(6)-Nd(1)-O(5)	48.93(4)
O(1)-Nd(1)-O(3)#2	127.35(5)	N(2)-Nd(1)-O(5)	113.94(5)
O(2)#1-Nd(1)-O(3)#2	84.40(5)	N(1)-Nd(1)-O(5)	94.77(5)
O(5)#1-Nd(1)-O(3)#2	96.20(6)	O(1)-Nd(1)-C(14)#2	101.24(6)
O(4)#2-Nd(1)-O(3)#2	52.04(5)	O(2)#1-Nd(1)-C(14)#2	106.31(6)
O(1)-Nd(1)-O(6)	71.44(5)	O(5)#1-Nd(1)-C(14)#2	89.20(6)
O(2)#1-Nd(1)-O(6)	103.77(6)	O(4)#2-Nd(1)-C(14)#2	25.93(6)
O(5)#1-Nd(1)-O(6)	123.29(5)	O(3)#2-Nd(1)-C(14)#2	26.11(6)
O(4)#2-Nd(1)-O(6)	128.59(5)	O(6)-Nd(1)-C(14)#2	141.10(5)
O(3)#2-Nd(1)-O(6)	140.45(5)	N(2)-Nd(1)-C(14)#2	55.64(10)
O(1)-Nd(1)-N(2)	83.74(6)	N(1)-Nd(1)-C(14)#2	167.12(5)
O(2)#1-Nd(1)-N(2)	134.62(6)	O(5)-Nd(1)-C(14)#2	70.98(5)
O(5)#1-Nd(1)-N(2)	151.41(5)	O(1)-Nd(1)-C(15)	76.15(6)
O(4)#2-Nd(1)-N(2)	73.29(5)	O(2)#1-Nd(1)-C(15)	88.08(6)
O(3)#2-Nd(1)-N(2)	81.43(6)	O(5)#1-Nd(1)-C(15)	101.62(5)
O(6)-Nd(1)-N(2)	65.18(5)	O(4)#2-Nd(1)-C(15)	143.40(5)
O(1)-Nd(1)-N(1)	135.31(6)	O(3)#2-Nd(1)-C(15)	157.66(5)
O(2)#1-Nd(1)-N(1)	73.11(6)	O(6)-Nd(1)-C(15)	23.95(5)
O(5)#1-Nd(1)-N(1)	145.76(6)	N(2)-Nd(1)-C(15)	89.09(6)
O(4)#2-Nd(1)-N(1)	116.07(5)	N(1)-Nd(1)-C(15)	80.37(5)
O(3)#2-Nd(1)-N(1)	77.32(6)	O(5)-Nd(1)-C(15)	24.99(5)
O(6)-Nd(1)-N(1)	68.69(5)	C(14)#2-Nd(1)-C(15)	164.16(6)
N(2)-Nd(1)-N(1)	61.78(6)		

Symmetry transformations used to generate equivalent atoms: #1 -x+1/2, -y+3/2, -z; #2 -x+1/2, y+1/2, -z+1/2; #3 -x+1/2, y-1/2, -z+1/2; #4 -x, y, -z-1/2.

**Table 8:** Selected bond lengths and bond angles of **1 h**.

Y(1)-O(5)	2.2662(15)	Y(1)-O(1)	2.4080(13)
Y(1)-O(4)#1	2.2983(13)	Y(1)-N(2)	2.5418(15)
Y(1)-O(3)#2	2.3225(13)	Y(1)-N(1)	2.6141(15)
Y(1)-O(6)#3	2.3602(15)	Y(1)-C(1)	2.7670(18)
Y(1)-O(2)	2.4016(13)		
O(5)-Y(1)-O(4)#1	73.25(5)	O(6)#3-Y(1)-N(2)	70.45(5)
O(5)-Y(1)-O(3)#2	79.97(6)	O(2)-Y(1)-N(2)	73.35(5)
O(4)#1-Y(1)-O(3)#2	130.88(5)	O(1)-Y(1)-N(2)	83.54(5)
O(5)-Y(1)-O(6)#3	125.93(7)	O(5)-Y(1)-N(1)	142.54(6)
O(4)#1-Y(1)-O(6)#3	90.33(6)	O(4)#1-Y(1)-N(1)	72.57(5)
O(3)#2-Y(1)-O(6)#3	73.14(5)	O(3)#2-Y(1)-N(1)	135.37(5)
O(5)-Y(1)-O(2)	78.70(6)	O(6)#3-Y(1)-N(1)	69.05(5)
O(4)#1-Y(1)-O(2)	135.32(5)	O(2)-Y(1)-N(1)	116.96(5)
O(3)#2-Y(1)-O(2)	75.26(5)	O(1)-Y(1)-N(1)	76.57(5)
O(6)#3-Y(1)-O(2)	134.34(6)	N(2)-Y(1)-N(1)	63.56(5)
O(5)-Y(1)-O(1)	89.23(7)	O(5)-Y(1)-C(1)	82.20(7)
O(4)#1-Y(1)-O(1)	91.05(5)	O(4)#1-Y(1)-C(1)	113.73(6)
O(3)#2-Y(1)-O(1)	129.40(4)	O(3)#2-Y(1)-C(1)	102.17(5)
O(6)#3-Y(1)-O(1)	143.43(5)	O(6)#3-Y(1)-C(1)	148.48(6)
O(2)-Y(1)-O(1)	54.13(5)	O(2)-Y(1)-C(1)	26.93(5)
O(5)-Y(1)-N(2)	149.86(6)	O(1)-Y(1)-C(1)	27.24(5)
O(4)#1-Y(1)-N(2)	135.87(5)	N(2)-Y(1)-C(1)	78.05(5)
O(3)#2-Y(1)-N(2)	82.21(5)	N(1)-Y(1)-C(1)	97.93(5)

Symmetry transformations used to generate equivalent atoms: #1 ×, -y+2, z-1/2; #2 -x+3/2, y-1/2, -z+3/2; #3 -x+3/2, -y+3/2, -z+1; #4 -x+3/2, y+1/2, -z+3/2; #5 ×, -y+2, z+1/2; #6 -x+2, y, -z+3/2.

## 2 Experimental design, materials, and methods

Ln-MOFs **1a-1h** were synthesized with similar procedure. 100 mg (0.396 mmol) H_2_ADA and 20 mL H_2_O were mixed in a 50 mL beaker, and adjusted to pH = 6 with 0.1 M NaOH solution. The ligand solution mixed with 20 mL water solution which contains 0.26 mmol Ln(NO_3_)_3_·6H_2_O. Then, 48 mg phen EtOH solution (20 mL) was added to the upward mixed solution, the reaction mixture was transferred to a bottle and sealed, reacted at 60 °C for three days. After cooling to room temperature in the oven, colorless crystals suitable for X-ray single crystal test were obtained by filtration, they were washed with 5 mL EtOH three times and air-dried.

Single crystal X-ray diffraction data was collected on a Bruker SMART 1000 CCD, with Mo-Ka radiation (Wavelength = 0.71073 Å) at room temperature. The structure was refined by full-matrix least-squares methods with SHELXL-97 module. Phase purity of bulk sample was determined by PXRD, using a DMAX2200VPC diffractometer, at 30 kV and 30 mA.
