# Supplementary material for: Structural data of thermostable 3D Ln-MOFs that based on flexible ligand of 1,3-adamantanediacetic acid
Source: Data Brief. 2018 Feb 3;17:689–97. doi: 10.1016/j.dib.2018.01.094 (PMC5854545; doi:10.1016/j.dib.2018.01.094)

checkCIF/PLATON report

Structure factors have been supplied for datablock(s) 1b

THIS REPORT IS FOR GUIDANCE ONLY. IF USED AS PART OF A REVIEW PROCEDURE FOR PUBLICATION, IT SHOULD NOT REPLACE THE EXPERTISE OF AN EXPERIENCED CRYSTALLOGRAPHIC REFEREE.

No syntax errors found. CIF dictionary Interpreting this report

Datablock: 1b

```
Bond precision:  C-C = 0.0066 A          Wavelength=0.71073

Cell:           a=23.2228(7)    b=11.5589(2)       c=24.2409(7)
                alpha=90        beta=120.102(4)     gamma=90
Temperature:    293 K

                Calculated                 Reported
Volume          5629.4(3)                  5629.4(3)
Space group     C 2/c                      C2/c
Hall group      -C 2yc                     ?
Moiety formula  C33 H35 Gd N2 O6           ?
Sum formula     C33 H35 Gd N2 O6           C33 H35 Gd N2 O6
Mr              712.88                     712.88
Dx,g cm-3       1.682                      1.682
Z               8                          8
Mu (mm-1)       2.407                      2.407
F000            2872.0                     2872.0
F000'           2871.73
h,k,lmax        27,13,28                   27,13,28
Nref            4964                       4954
Tmin,Tmax       0.618,0.697                0.619,0.714
Tmin'           0.583

Correction method= # Reported T Limits: Tmin=0.619 Tmax=0.714
AbsCorr = MULTI-SCAN

Data completeness= 0.998          Theta(max)= 25.010

R(reflections)= 0.0280( 4665)     wR2(reflections)= 0.0930( 4954)

S = 1.003                  Npar= 380
```

The following ALERTS were generated. Each ALERT has the format
test-name_ALERT_alert-type_alert-level.
Click on the hyperlinks for more details of the test.

Alert level C

```
ABSTY02_ALERT_1_C  An _exptl_absorpt_correction_type has been given without
            a literature citation. This should be contained in the
            _exptl_absorpt_process_details field.
            Absorption correction given as multi-scan
CRYSC01_ALERT_1_C The word below has not been recognised as a standard
            identifier.
            yellowish
CRYSC01_ALERT_1_C No recognised colour has been given for crystal colour.
PLAT213_ALERT_2_C Atom C21             has ADP max/min Ratio .....        3.4 prolat
PLAT213_ALERT_2_C Atom C22             has ADP max/min Ratio .....        3.8 prolat
PLAT213_ALERT_2_C Atom C30             has ADP max/min Ratio .....        3.4 prolat
PLAT220_ALERT_2_C Non-Solvent Resd 1   C   Ueq(max)/Ueq(min) Range        5.7 Ratio
PLAT222_ALERT_3_C Non-Solvent Resd 1   H Uiso(max)/Uiso(min) Range        5.5 Ratio
PLAT910_ALERT_3_C Missing # of FCF Reflection(s) Below Theta(Min)           9 Note
```

Alert level G

```
PLAT004_ALERT_5_G Polymeric Structure Found with Maximum Dimension          3 Info
PLAT005_ALERT_5_G No Embedded Refinement Details found  in the CIF     Please Do !
PLAT083_ALERT_2_G SHELXL Second Parameter in WGHT  Unusually Large      33.72 Why ?
PLAT093_ALERT_1_G No s.u.'s on H-positions, Refinement Reported as      mixed Check
PLAT158_ALERT_4_G The Input Unitcell is NOT Standard/Reduced .....     Please Check
PLAT199_ALERT_1_G Reported _cell_measurement_temperature ..... (K)        293 Check
PLAT200_ALERT_1_G Reported   _diffrn_ambient_temperature ..... (K)        293 Check
PLAT300_ALERT_4_G Atom Site Occupancy of H19A    is Constrained at        0.5 Check
PLAT300_ALERT_4_G Atom Site Occupancy of H19B    is Constrained at        0.5 Check
PLAT300_ALERT_4_G Atom Site Occupancy of H22A    is Constrained at        0.5 Check
PLAT300_ALERT_4_G Atom Site Occupancy of H22B    is Constrained at        0.5 Check
PLAT333_ALERT_2_G Check Large Av C6-Ring C-C Dist. C26    -C31           1.42 Ang.
PLAT367_ALERT_2_G Long?   C(sp?)-C(sp?) Bond  C17    -   C19    ..       1.54 Ang.
PLAT710_ALERT_4_G Delete 1-2-3 or 2-3-4 Linear Torsion Angle ... #         33 Do !
            O2  -GD1 -C15 -C16  -132.00  2.00   5.656   1.555   1.555   1.555
PLAT710_ALERT_4_G Delete 1-2-3 or 2-3-4 Linear Torsion Angle ... #         34 Do !
            O1  -GD1 -C15 -C16    87.00  2.00   1.555   1.555   1.555   1.555
PLAT710_ALERT_4_G Delete 1-2-3 or 2-3-4 Linear Torsion Angle ... #         35 Do !
            O6  -GD1 -C15 -C16   159.00  2.00   5.656   1.555   1.555   1.555
PLAT710_ALERT_4_G Delete 1-2-3 or 2-3-4 Linear Torsion Angle ... #         36 Do !
            O4  -GD1 -C15 -C16  -106.00  2.00   8.556   1.555   1.555   1.555
PLAT710_ALERT_4_G Delete 1-2-3 or 2-3-4 Linear Torsion Angle ... #         37 Do !
            O3  -GD1 -C15 -C16    14.00  2.00   8.556   1.555   1.555   1.555
PLAT710_ALERT_4_G Delete 1-2-3 or 2-3-4 Linear Torsion Angle ... #         38 Do !
            O5  -GD1 -C15 -C16   -45.20  1.90   1.555   1.555   1.555   1.555
PLAT710_ALERT_4_G Delete 1-2-3 or 2-3-4 Linear Torsion Angle ... #         39 Do !
            N1  -GD1 -C15 -C16   -47.00  2.00   1.555   1.555   1.555   1.555
PLAT710_ALERT_4_G Delete 1-2-3 or 2-3-4 Linear Torsion Angle ... #         40 Do !
            N2  -GD1 -C15 -C16    14.00  2.00   1.555   1.555   1.555   1.555
PLAT710_ALERT_4_G Delete 1-2-3 or 2-3-4 Linear Torsion Angle ... #         41 Do !
            O6  -GD1 -C15 -C16   138.00  2.00   1.555   1.555   1.555   1.555
PLAT710_ALERT_4_G Delete 1-2-3 or 2-3-4 Linear Torsion Angle ... #         42 Do !
            C14 -GD1 -C15 -C16   -69.00  2.00   8.556   1.555   1.555   1.555
PLAT710_ALERT_4_G Delete 1-2-3 or 2-3-4 Linear Torsion Angle ... #         43 Do !
            GD1 -GD1 -C15 -C16   152.00  2.00   5.656   1.555   1.555   1.555
PLAT710_ALERT_4_G Delete 1-2-3 or 2-3-4 Linear Torsion Angle ... #         46 Do !
            GD1 -C15 -C16 -C17   -47.00  2.00   1.555   1.555   1.555   1.555
PLAT710_ALERT_4_G Delete 1-2-3 or 2-3-4 Linear Torsion Angle ... #        228 Do !
            C12 -C13 -C14 -GD1  -112.00  7.00   1.555   1.555   1.555   8.455
PLAT764_ALERT_4_G Overcomplete CIF Bond List Detected (Rep/Expd) .       1.20 Ratio
PLAT774_ALERT_1_G Suspect X-Y Bond in CIF:   Gd1    --  Gd1     ..       4.04 Ang.
PLAT793_ALERT_4_G The Model has Chirality at C5      (Centro SPGR)          S Verify
PLAT793_ALERT_4_G The Model has Chirality at C7      (Centro SPGR)          R Verify
```

```
PLAT899_ALERT_4_G SHELXL97   is Deprecated and Succeeded by SHELXL       2014 Note
PLAT909_ALERT_3_G Percentage of Observed Data at Theta(Max) Still          91 % Note
PLAT978_ALERT_2_G Number C-C Bonds with Positive Residual Density.         11 Note
```

```
  0 ALERT level A = Most likely a serious problem - resolve or explain
  0 ALERT level B = A potentially serious problem, consider carefully
  9 ALERT level C = Check. Ensure it is not caused by an omission or oversight
 33 ALERT level G = General information/check it is not something unexpected

  7 ALERT type 1 CIF construction/syntax error, inconsistent or missing data
  8 ALERT type 2 Indicator that the structure model may be wrong or deficient
  3 ALERT type 3 Indicator that the structure quality may be low
 22 ALERT type 4 Improvement, methodology, query or suggestion
  2 ALERT type 5 Informative message, check
```

It is advisable to attempt to resolve as many as possible of the alerts in all categories. Often the minor alerts point to easily fixed oversights, errors and omissions in your CIF or refinement strategy, so attention to these fine details can be worthwhile. In order to resolve some of the more serious problems it may be necessary to carry out additional measurements or structure refinements. However, the purpose of your study may justify the reported deviations and the more serious of these should normally be commented upon in the discussion or experimental section of a paper or in the "special_details" fields of the CIF. checkCIF was carefully designed to identify outliers and unusual parameters, but every test has its limitations and alerts that are not important in a particular case may appear. Conversely, the absence of alerts does not guarantee there are no aspects of the results needing attention. It is up to the individual to critically assess their own results and, if necessary, seek expert advice.

Publication of your CIF in IUCr journals

A basic structural check has been run on your CIF. These basic checks will be run on all CIFs submitted for publication in IUCr journals (*Acta Crystallographica*, *Journal of Applied Crystallography*, *Journal of Synchrotron Radiation*); however, if you intend to submit to *Acta Crystallographica Section C* or *E* or *IUCrData*, you should make sure that full publication checks are run on the final version of your CIF prior to submission.

Publication of your CIF in other journals

Please refer to the *Notes for Authors* of the relevant journal for any special instructions relating to CIF submission.

PLATON version of 27/03/2017; check.def file version of 24/03/2017

Datablock 1b - ellipsoid plot

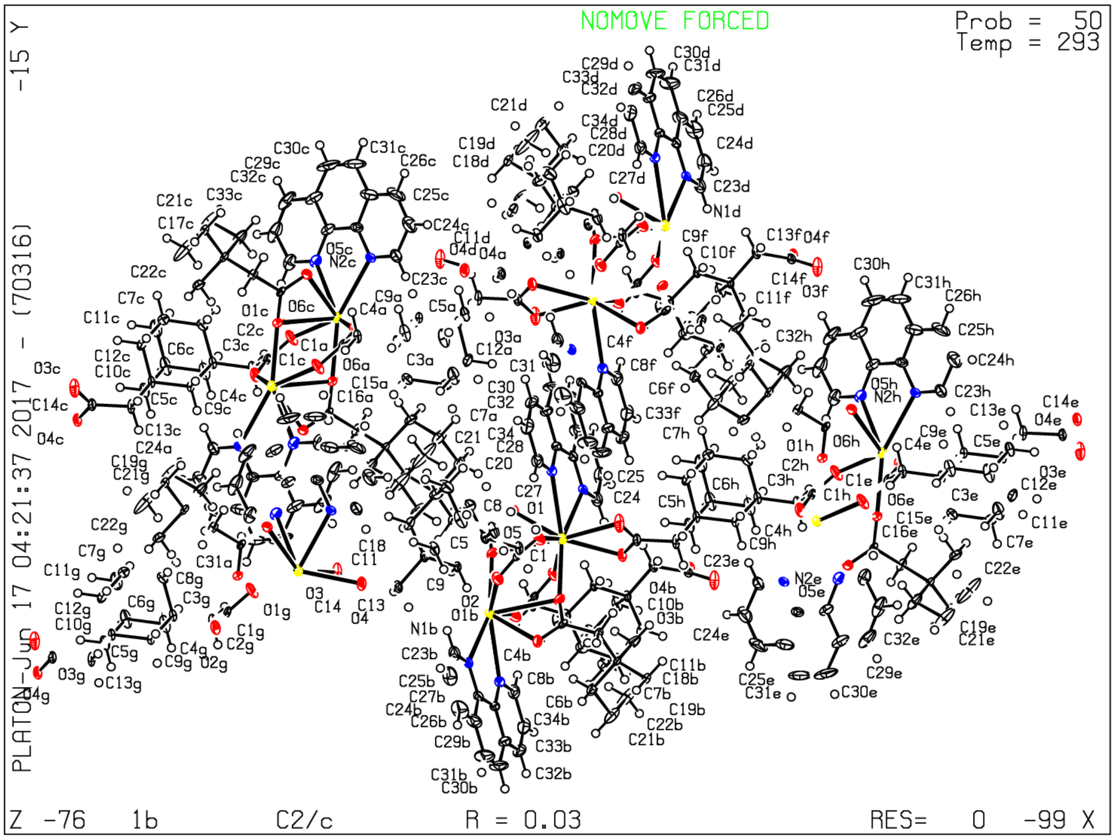

Supplement: Supplementary file 2 — Supplementary material [file mmc2.pdf]
