# Supplementary material for: Structural data of thermostable 3D Ln-MOFs that based on flexible ligand of 1,3-adamantanediacetic acid
Source: Data Brief. 2018 Feb 3;17:689–97. doi: 10.1016/j.dib.2018.01.094 (PMC5854545; doi:10.1016/j.dib.2018.01.094)

checkCIF/PLATON report

Structure factors have been supplied for datablock(s) 1e

THIS REPORT IS FOR GUIDANCE ONLY. IF USED AS PART OF A REVIEW PROCEDURE FOR PUBLICATION, IT SHOULD NOT REPLACE THE EXPERTISE OF AN EXPERIENCED CRYSTALLOGRAPHIC REFEREE.

No syntax errors found. CIF dictionary Interpreting this report

Datablock: 1e

Bond precision: C-C = 0.0036 A Wavelength=0.71073

Cell:	a=23.2395(7)	b=11.5478(2)	c=23.7207(7)
	alpha=90	beta=117.871(4)	gamma=90
Temperature:	293 K		

	Calculated	Reported
Volume	5627.4(3)	5627.4(3)
Space group	C 2/c	C2/c
Hall group	-C 2yc	?
Moiety formula	C33 H35 Ce N2 O6	?
Sum formula	C33 H35 Ce N2 O6	C4 H4.24 Ce0.12 N0.24 O0.73
Mr	695.75	84.33
Dx,g cm-3	1.642	1.642
Z	8	66
Mu (mm-1)	1.668	1.668
F000	2824.0	2824.0
F000’	2823.08	
h,k,lmax	27,13,28	27,13,28
Nref	4961	4950
Tmin,Tmax	0.756,0.819	0.671,0.825
Tmin’	0.648	

Correction method= # Reported T Limits: Tmin=0.671 Tmax=0.825
AbsCorr = MULTI-SCAN

Data completeness= 0.998 Theta(max)= 25.010

R(reflections)= 0.0177(4684) wR2(reflections)= 0.0430(4950)

S = 1.009 Npar= 380

The following ALERTS were generated. Each ALERT has the format
test-name_ALERT_alert-type_alert-level.
Click on the hyperlinks for more details of the test.

Alert level B

```
PLAT910_ALERT_3_B Missing # of FCF Reflection(s) Below Theta(Min)         11 Note
```

Author Response: Theta min=3.8860 deg. The theta value of the missing reflections are less than 3.8760 deg. and therefore reflections were excluded.

Alert level C

```
ABSTY02_ALERT_1_C  An _exptl_absorpt_correction_type has been given without
            a literature citation. This should be contained in the
            _exptl_absorpt_process_details field.
            Absorption correction given as multi-scan
CRYSC01_ALERT_1_C The word below has not been recognised as a standard
            identifier.
            yellowish
CRYSC01_ALERT_1_C No recognised colour has been given for crystal colour.
PLAT220_ALERT_2_C Non-Solvent Resd 1   C   Ueq(max)/Ueq(min) Range        4.0 Ratio
```

Alert level G

```
CELLZ01_ALERT_1_G Difference between formula and atom_site contents detected.
CELLZ01_ALERT_1_G ALERT: Large difference may be due to a
            symmetry error - see SYMMG tests
           From the CIF: _cell_formula_units_Z   66
           From the CIF: _chemical_formula_sum  C4 H4.24 Ce0.12 N0.24 O0.73
           TEST: Compare cell contents of formula and atom_site data

           atom    Z*formula  cif sites diff
           C        264.00    264.00    0.00
           H        279.84    280.00   -0.16
           Ce         7.92      8.00   -0.08
           N         15.84     16.00   -0.16
           O         48.18     48.00    0.18
PLAT004_ALERT_5_G Polymeric Structure Found with Maximum Dimension         3 Info
PLAT005_ALERT_5_G No Embedded Refinement Details found  in the CIF     Please Do !
PLAT045_ALERT_1_G Calculated and Reported Z Differ by a Factor ...       0.12 Check
PLAT083_ALERT_2_G SHELXL Second Parameter in WGHT  Unusually Large       8.95 Why ?
PLAT093_ALERT_1_G No s.u.'s on H-positions, Refinement Reported as      mixed Check
PLAT199_ALERT_1_G Reported _cell_measurement_temperature ..... (K)        293 Check
PLAT200_ALERT_1_G Reported   _diffrn_ambient_temperature ..... (K)        293 Check
PLAT300_ALERT_4_G Atom Site Occupancy of H18A    is Constrained at        0.5 Check
PLAT300_ALERT_4_G Atom Site Occupancy of H18B    is Constrained at        0.5 Check
PLAT300_ALERT_4_G Atom Site Occupancy of H22A    is Constrained at        0.5 Check
PLAT300_ALERT_4_G Atom Site Occupancy of H22B    is Constrained at        0.5 Check
PLAT367_ALERT_2_G Long?   C(sp?)-C(sp?) Bond  C17    -   C18    ..       1.54 Ang.
PLAT367_ALERT_2_G Long?   C(sp?)-C(sp?) Bond  C20    -   C22    ..       1.51 Ang.
PLAT710_ALERT_4_G Delete 1-2-3 or 2-3-4 Linear Torsion Angle ... #        108 Do !
            O1  -CE1 -C15 -C16   130.60  1.10   1.555   1.555   1.555   1.555
PLAT710_ALERT_4_G Delete 1-2-3 or 2-3-4 Linear Torsion Angle ... #        109 Do !
            O2  -CE1 -C15 -C16   -88.30  1.10   7.656   1.555   1.555   1.555
PLAT710_ALERT_4_G Delete 1-2-3 or 2-3-4 Linear Torsion Angle ... #        110 Do !
            O6  -CE1 -C15 -C16  -160.10  1.10   7.656   1.555   1.555   1.555
```

```
PLAT710_ALERT_4_G Delete 1-2-3 or 2-3-4 Linear Torsion Angle ... #       111 Do !
            O3  -CE1 -C15 -C16   104.90  1.10   4.656   1.555   1.555   1.555
PLAT710_ALERT_4_G Delete 1-2-3 or 2-3-4 Linear Torsion Angle ... #       112 Do !
            O4  -CE1 -C15 -C16   -15.30  1.20   4.656   1.555   1.555   1.555
PLAT710_ALERT_4_G Delete 1-2-3 or 2-3-4 Linear Torsion Angle ... #       113 Do !
            O5  -CE1 -C15 -C16    43.90  1.10   1.555   1.555   1.555   1.555
PLAT710_ALERT_4_G Delete 1-2-3 or 2-3-4 Linear Torsion Angle ... #       114 Do !
            N1  -CE1 -C15 -C16    45.80  1.10   1.555   1.555   1.555   1.555
PLAT710_ALERT_4_G Delete 1-2-3 or 2-3-4 Linear Torsion Angle ... #       115 Do !
            N2  -CE1 -C15 -C16   -14.90  1.10   1.555   1.555   1.555   1.555
PLAT710_ALERT_4_G Delete 1-2-3 or 2-3-4 Linear Torsion Angle ... #       116 Do !
            O6  -CE1 -C15 -C16  -139.00  1.20   1.555   1.555   1.555   1.555
PLAT710_ALERT_4_G Delete 1-2-3 or 2-3-4 Linear Torsion Angle ... #       117 Do !
            C14 -CE1 -C15 -C16    67.20  1.20   4.656   1.555   1.555   1.555
PLAT710_ALERT_4_G Delete 1-2-3 or 2-3-4 Linear Torsion Angle ... #       118 Do !
            CE1 -CE1 -C15 -C16  -153.30  1.10   7.656   1.555   1.555   1.555
PLAT710_ALERT_4_G Delete 1-2-3 or 2-3-4 Linear Torsion Angle ... #       121 Do !
            CE1 -C15 -C16 -C17    48.60  1.20   1.555   1.555   1.555   1.555
PLAT710_ALERT_4_G Delete 1-2-3 or 2-3-4 Linear Torsion Angle ... #       227 Do !
            C12 -C13 -C14 -CE1  -116.00  4.00   1.555   1.555   1.555   4.646
PLAT764_ALERT_4_G Overcomplete CIF Bond List Detected (Rep/Expd) .      1.20 Ratio
PLAT774_ALERT_1_G Suspect X-Y Bond in CIF:   Ce1    --  Ce1     ..      4.04 Ang.
PLAT793_ALERT_4_G The Model has Chirality at C5      (Centro SPGR)         R Verify
PLAT793_ALERT_4_G The Model has Chirality at C7      (Centro SPGR)         S Verify
PLAT793_ALERT_4_G The Model has Chirality at C20     (Centro SPGR)         S Verify
PLAT899_ALERT_4_G SHELXL97   is Deprecated and Succeeded by SHELXL      2014 Note
PLAT909_ALERT_3_G Percentage of Observed Data at Theta(Max) Still         91 % Note
PLAT961_ALERT_5_G Dataset Contains no Negative Intensities .......    Please Check
PLAT978_ALERT_2_G Number C-C Bonds with Positive Residual Density.        16 Note
```

---

0 **ALERT level A** = Most likely a serious problem - resolve or explain
1 **ALERT level B** = A potentially serious problem, consider carefully
4 **ALERT level C** = Check. Ensure it is not caused by an omission or oversight
37 **ALERT level G** = General information/check it is not something unexpected

10 ALERT type 1 CIF construction/syntax error, inconsistent or missing data
5 ALERT type 2 Indicator that the structure model may be wrong or deficient
2 ALERT type 3 Indicator that the structure quality may be low
22 ALERT type 4 Improvement, methodology, query or suggestion
3 ALERT type 5 Informative message, check

It is advisable to attempt to resolve as many as possible of the alerts in all categories. Often the minor alerts point to easily fixed oversights, errors and omissions in your CIF or refinement strategy, so attention to these fine details can be worthwhile. In order to resolve some of the more serious problems it may be necessary to carry out additional measurements or structure refinements. However, the purpose of your study may justify the reported deviations and the more serious of these should normally be commented upon in the discussion or experimental section of a paper or in the "special_details" fields of the CIF. checkCIF was carefully designed to identify outliers and unusual parameters, but every test has its limitations and alerts that are not important in a particular case may appear. Conversely, the absence of alerts does not guarantee there are no aspects of the results needing attention. It is up to the individual to critically assess their own results and, if necessary, seek expert advice.

Publication of your CIF in IUCr journals

A basic structural check has been run on your CIF. These basic checks will be run on all CIFs submitted for publication in IUCr journals (*Acta Crystallographica*, *Journal of Applied Crystallography*, *Journal of Synchrotron Radiation*); however, if you intend to submit to *Acta Crystallographica Section C* or *E* or *IUCrData*, you should make sure that full publication checks are run on the final version of your CIF prior to submission.

Publication of your CIF in other journals

Please refer to the *Notes for Authors* of the relevant journal for any special instructions relating to CIF submission.

PLATON version of 27/03/2017; check.def file version of 24/03/2017

Datablock 1e - ellipsoid plot

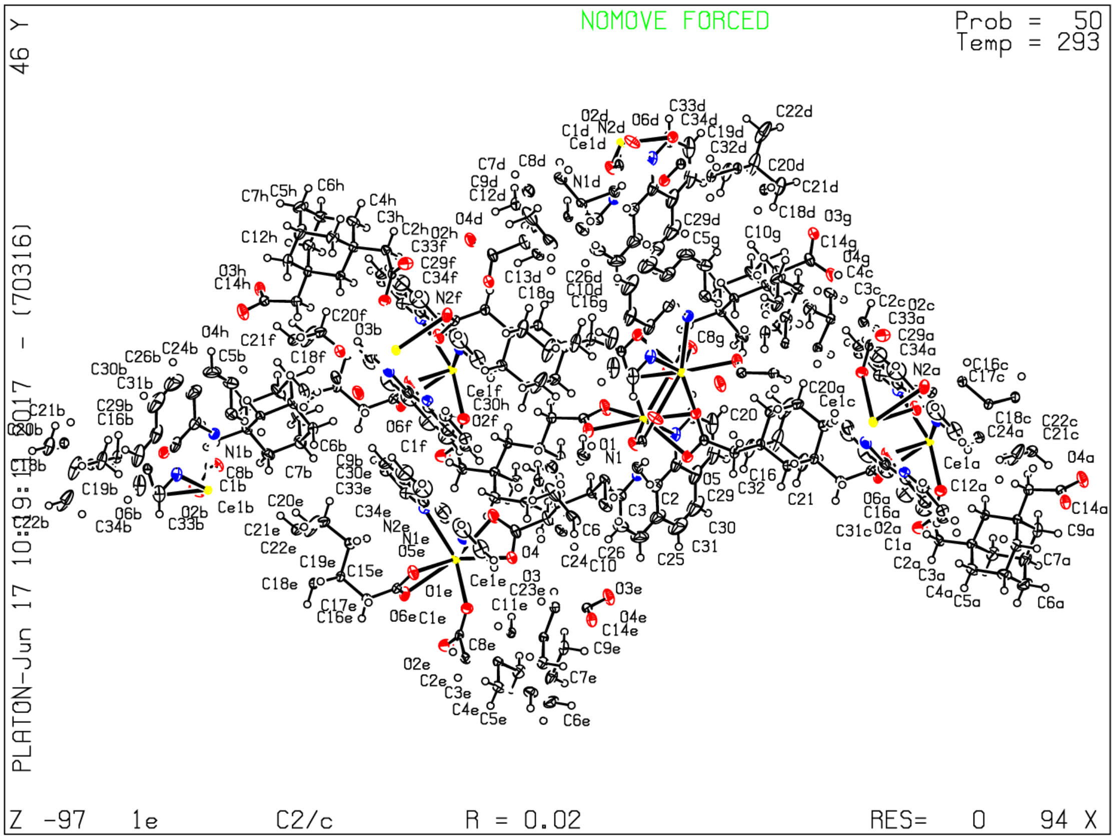

Supplement: Supplementary file 4 — Supplementary material [file mmc4.pdf]
